# Cryo-cooled silicon crystal monochromators: a study of power load, temperature and deformation

**DOI:** 10.1107/S160057752200039X

**Published:** 2022-02-08

**Authors:** Hossein Khosroabadi, Lucia Alianelli, Daniel G. Porter, Steve Collins, Kawal Sawhney

**Affiliations:** a Diamond Light Source, Diamond House Harwell Science and Innovation Campus, Oxfordshire OX11 0DE, United Kingdom

**Keywords:** cryo-cooled Si monochromator, deformation modelling, lensing effect, cooling design

## Abstract

Crystal monochromators are often the primary optics in hard X-ray synchrotron beamlines. Management of power load is central to their design. Strict requirements on stability and deformation are to be met, as new-generation synchrotron sources deliver brighter beams of X-rays. This article sets out to illustrate an overall picture of the deformation caused by heat load in a cryo-cooled Si crystal monochromator using first principles. A theoretical model has been developed to predict the temperature distribution and surface deformation by applying intrinsic properties of Si material and the cooling system parameters. The model explains the universal behaviour of crystal slope error versus absorbed power; it has been benchmarked against experimental data and used to interpret finite-element analysis of cryogenically cooled crystals.

## Abstract 2

The heat-load effects on cryo-cooled Si crystals in synchrotron beamlines are investigated.

## 1. Introduction

Cryo-cooled silicon crystals have been used for decades as monochromators in hard X-ray synchrotron beamlines. Relatively large thermal conductivity at low temperature and zero linear thermal expansion coefficient at temperatures *T*_zero_ ≃ 125 K result in low crystal deformation compared with other perfect crystal materials, or to water-cooled silicon (Chumakov *et al.*, 2004 ▸; Zhang *et al.*, 2003 ▸; Marot *et al.*, 1992 ▸; Yates *et al.*, 2010 ▸). Well designed cryo-cooling systems successfully minimize the surface deformation even under high power load from intense undulator sources. However, on-going upgrades of synchrotron photon sources require further improvements of optical components. The aim is to conserve the natural collimation and brightness of synchrotron beams in lower-emittance machines (Huang *et al.*, 2014 ▸; Brumund *et al.*, 2021 ▸; Zhang *et al.*, 2013 ▸).

Considerable increase of incident power *P* and power density *P*_d_ on monochromators is currently observed on some hard X-ray beamlines at Diamond Light Source (DLS), equipped with new higher-magnetic-field undulators. Power will increase further with the new Diamond-II machine, when the ring energy is increased from 3 to 3.5 GeV (Diamond-II Conceptual Design Report: https://www.diamond.ac.uk/Home/About/Vision/Diamond-II.html). Effective control of power is fundamental and can be accomplished by use of smaller primary slits and filter materials. An optimized crystal cooling design will reduce optics deformations, prevent changes in diffracted beam wavefront and improve focusing.

Finite-element analysis (FEA) has been widely used to investigate thermal deformation of beamline optics such as mirrors and monochromators (Zhang *et al.*, 2003 ▸, 2013 ▸; Yates *et al.*, 2010 ▸; Dolbnya *et al.*, 2019 ▸; Cheng *et al.*, 2015 ▸). It can be a time-consuming analysis, as the predicted crystal deformation varies considerably with changing scenarios, such as incident power and cooling parameters. FEA is a powerful tool in the design of cooling systems, with the caveat that simulations may need interpretation depending on the assumptions and parameters used. For instance, in the indirect cooling geometry, contact conductance between crystal and copper cooling block is not easily determined, and FEA provides a wide range of slope errors depending on input values. *In situ* surface deformation measurements (Kazimirov *et al.*, 2007 ▸, 2008 ▸; Revesz *et al.*, 2007 ▸; Rutishauser *et al.*, 2013 ▸) require specialized set-ups, which are not readily available or feasible for any monochromator system.

A simplified model of temperature spatial distribution and deformation is presented in the first part of the article. It explains the properties of cryo-cooled Si crystals which are observed experimentally and predicted by FEA; therefore, it can be used for an initial assessment of cooling requirements. An experimental study of the I16 beamline monochromator (Collins *et al.*, 2010 ▸) at DLS is described in the second part of the article. The model reproduces the experimental data, therefore showing that, in combination with FEA, it is a simple and intuitive tool to design cooling of advanced synchrotron optics.

## 2. Theoretical study

### 2.1. Modelling

Temperature and crystal deformation as a function of time, power and power density are presented in this section. Solutions for the principal quantities such as *T*_b_, the crystal body temperature, *T*_p_, the peak surface temperature, *T*_a_, the temperature at the beam footprint margin, and Δ*L*, the thermal strain are derived in Appendix *A* [App appa].

**Figure 1:**
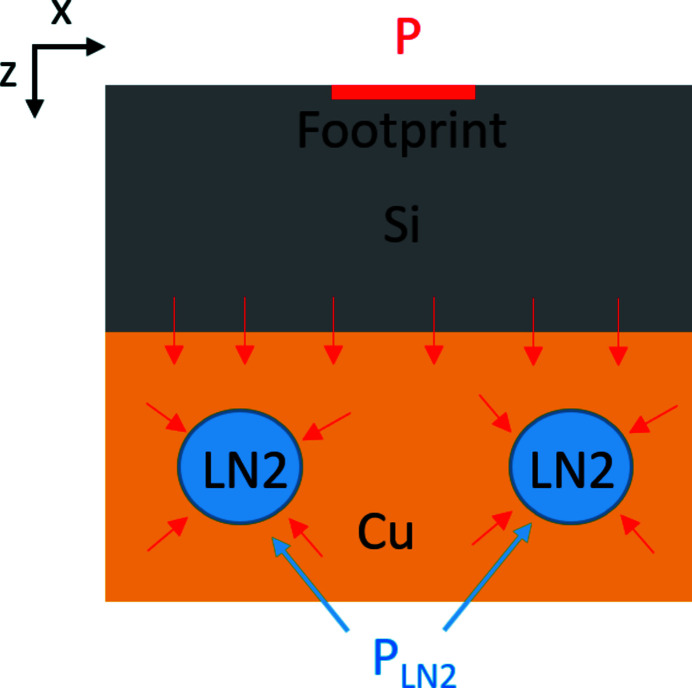
Schematic view of the indirect cryo-cooled monochromator. The red arrows show heat flow from the crystal to the cooling LN2 channels.

The model, schematically illustrated in Fig. 1 ▸, is valid for an indirectly cryo-cooled Si crystal but can be extended to the direct cooling geometry. Whilst *T*_b_ stays nearly constant, the temperature at the beam footprint area evolves rapidly (Zhang *et al.*, 2013 ▸) when this area is much smaller than the crystal surface.

The following coupled differential equations describe the temporal evolution of the average temperature of the cooling copper block *T*_Cu_ and Si crystal *T*,

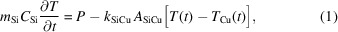




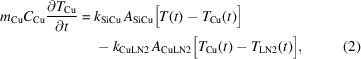

where *m*_Si(Cu)_ and *C*_Si(Cu)_ are the mass and specific heat capacity of Si (Cu); *k*_SiCu_ and *A*_SiCu_ are the conductance and area of the Si–Cu contact; similar symbols denote Cu and liquid nitro­gen (LN2). The analytical solution for *T*(*t*) at constant incident power and LN2 temperature is

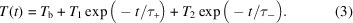

Expressions for *T*_b_, τ_+_ and τ_−_ are derived in Appendix *A* [App appa]. Crystal temperature increases exponentially with τ_+_ and τ_−_ time scales, until it reaches an asymptotic value *T*_b_. For a constant *T*_Cu_, only the first exponential term remains in equation (3).

Heat due to absorbed power is transferred with radial symmetry in the crystal, in good approximation. Constant temperature layers at a distance *r* from the centre of the beam footprint can be defined (Zhang *et al.*, 2003 ▸; Yates *et al.*, 2010 ▸). The relationship between radial power and crystal temperature gradient ∂*T*/∂*r* can therefore be expressed as

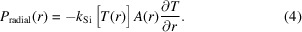

Here, *k*_Si_ and *A* are the Si thermal conductivity and area of the layer located at distance *r*. *T*(*r*) can be obtained by analytical integration for the circular footprint case, or numerically for the elliptical case. Large changes in *A* and *k*_Si_ with *r* lead to fast convergence of *T*(*r*) to *T*_b_ at distances 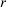 ≃ 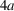 , where *a* is the radius of the beam footprint. A numerical solution can be determined for any power distribution. The solutions for uniform power density distribution, a good approximation for undulator beams at third-generation sources, are derived in the Appendix *A* [App appa].

Crystal surface deformation, for the stretching and bending components, is calculated *via* the following integrals,

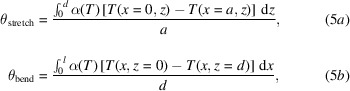

where α(*T*) denotes the Si linear expansion coefficient, *d* ≃ 2*a* is the integration depth and *l* is the length over which the crystal temperature is varying considerably.

Stretching is the result of the crystal freely expanding perpendicularly to the beam footprint. Since the much larger crystal bulk, at temperature *T*_b_, does not expand freely along the surface, the region near the footprint is subject to strong bending. This is often the dominant contribution to deformation, as temperature gradients are higher along depth. Approximate values for the slope error θ are derived by assuming constant temperature at the footprint and within a crystal depth *d* ≃ 2*a*. The bending term can be calculated *via* the thermal strain,

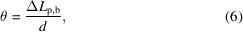

where

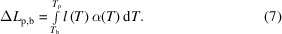




### 2.2. Discussion and applications

The model is valid under the following assumptions: the beam footprint is smaller than the size of the crystal; power distribution has circular or elliptical symmetry; mechanical stress or other forces can be ignored; and the crystal is free to expand perpendicular to the surface. Temperature *T*_b_ should be determined either experimentally or from equation (9).

Use of the model has allowed: verification and interpretation of measurements and FEA data; estimation of slope errors for a wide range of incident beam power, power density and footprints (or Bragg angles); assessment of existing crystal cooling designs; preliminarily design of new cooling systems. Determination of the LN2 flow rate and precise geometry of the cooling channels are outside of the scope of this study. As the model has so far provided accuracy of order 10% to 20%, compared with FEA or experimental data at DLS, the final monochromator design should rely on FEA.

**Figure 2:**
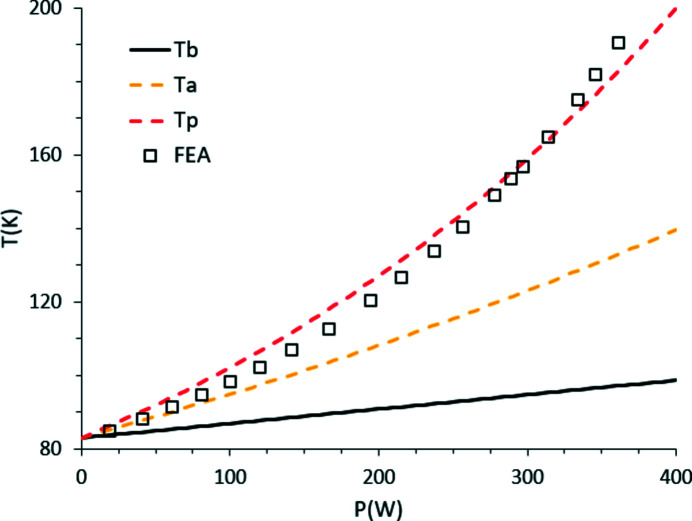
*T*_a_, *T*_p_ calculated from equations (14*a*) and (14*b*) and compared with FEA results (Zhang *et al.*, 2013 ▸). We have used *a* = 1.7 mm.

Comparison with the published FEA temperature data from a crystal monochromator at the ESRF (Zhang *et al.*, 2013 ▸) is presented in Fig. 2 ▸. We have used equations (14*a*) and (14*b*), with *T*_b_ extrapolated from the published data. Good agreement is observed, despite the approximations used to derive these solutions.

**Figure 3:**
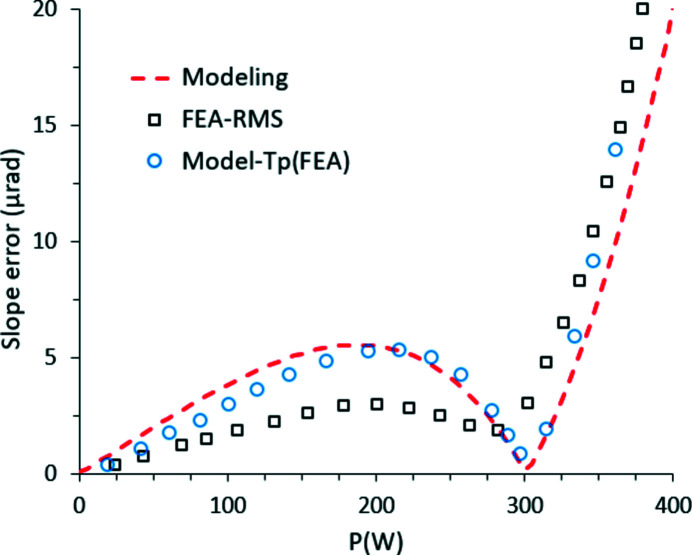
Slope error calculation from equations (6) and (7) (red dashes) compared with FEA results of Zhang *et al.* (2013 ▸). Blue circles are obtained from *T*_p_ values extrapolated from FEA.

Slope error values, as obtained from equations (6) and (7), are plotted next to the published data (Zhang *et al.*, 2013 ▸) in Fig. 3 ▸. Data trends are similar and deviation from FEA results are due to the complex dependence of the modelled phenomena on power distribution, total power, footprint, contact conductance and cooling design details.

**Figure 4:**
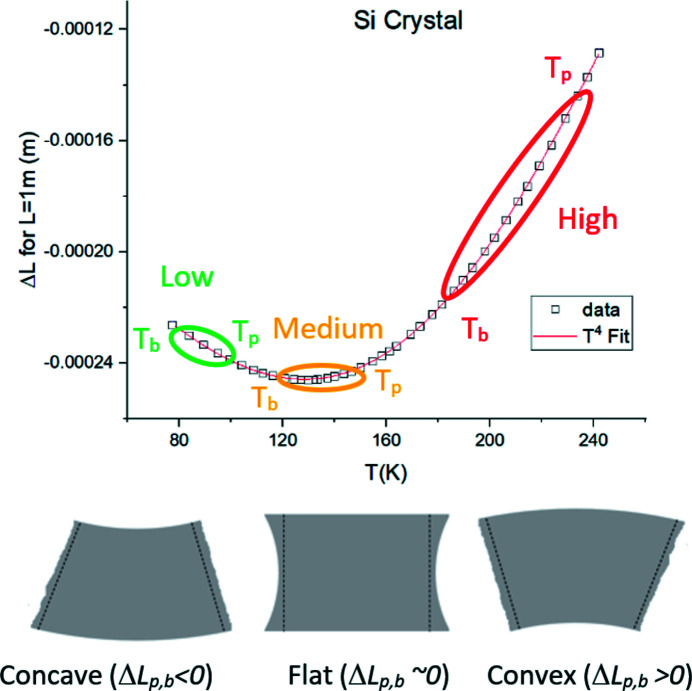
Top: length expansion of Si (squares) fitted by a *T* ^4^ polynomial (red line). The ellipses show the location of *T*_p_ and *T*_b_ on the graph for three different regions of low, medium and high power. Bottom: schematic shape of the crystal deformation from the concave to convex regime for the three power ranges.

The mechanism underlying the strongly non-linear crystal deformation is illustrated schematically in Fig. 4 ▸. The bending contribution causes three possible types of deformation: from concave shape at low power, to flat, and finally to highly convex at high power. This behaviour is caused by the unique properties of the Si thermal expansion coefficient and is universally observed in cryo-cooled Si crystals subject to heat load. The shape and amplitude of the deformation are determined by the characteristic temperatures (*T*_b_ and *T*_p_) of a given crystal, in each scenario of power load and cooling. Efforts to keep the temperature at a beam footprint close to *T*_zero_ do not result in minimum deformation. Deformation can be minimized, when temperatures *T*_b_ and *T*_p_ are symmetrical relative to *T*_zero_. This can explain the FEA result of a minimum surface deformation at *T*_p_ ≃ 150 K and *T*_b_ ≃ 95 K (Zhang *et al.*, 2013 ▸).

Further accuracy is obtained solving equation (4) numerically. Details such as crystal shape and size can be ignored, under the usual assumption of the crystal being much larger than the beam footprint. Crystal stress can be calculated, if required, by including mechanical properties of Si such as bulk modulus tensor and Poisson ratio. Slope error amplitude is calculated for *d* = 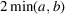 and over the entire footprint area. Calculations have been cross-checked again with several FEA studies at DLS.

**Figure 5:**
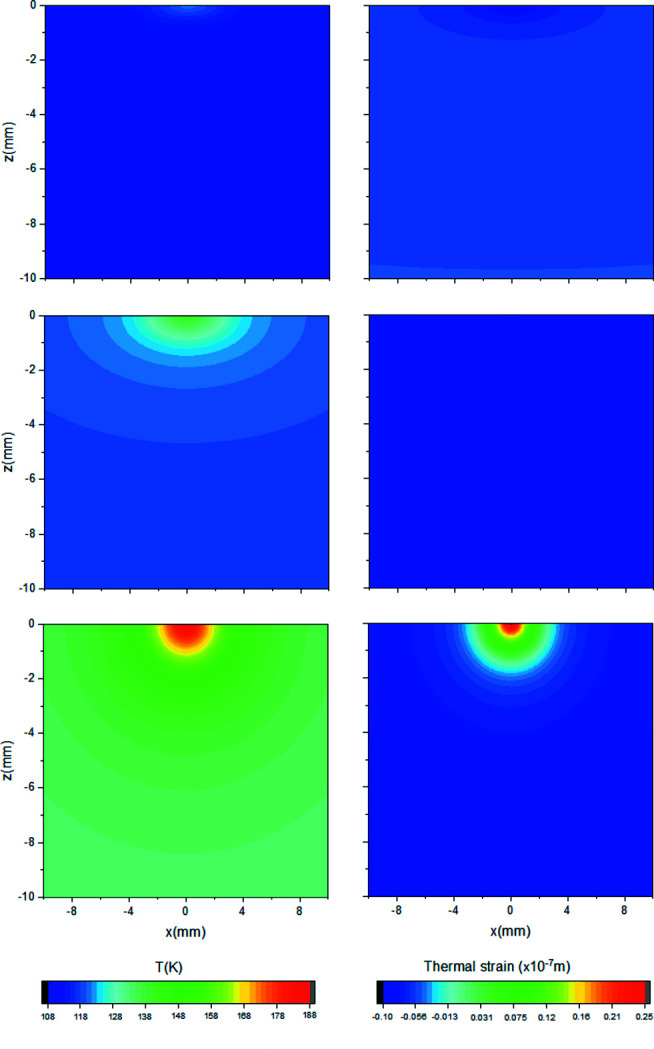
I16 Si crystal temperature and thermal strain calculated for different values of primary slit sizes, corresponding to *P* = 69 W, 116 W and 207 W (top to bottom) at fixed photon energy *E* = 3.5 keV. Left: temperature distribution. Right: thermal strain relative to 97 K (or *P* = 0).

Numerical models of temperature and thermal strain for the I16 crystal monochromator at photon energy *E* = 3.5 keV and varying incident power (*P* = 69 W, 116 W, 207 W) are shown in Fig. 5 ▸. The respective calculated temperature values are: *T*_b_ = 108 K, 116 K and 132 K; *T*_p_ = 121 K, 141 K and 188 K. These examples illustrate the three different temperature scenarios, corresponding to the low, medium and high power: (*T*_b_, *T*_p_) < *T*_zero_; *T*_b_ < *T*_zero_ < *T*_p_; (*T*_b_, *T*_p_) > *T*_zero_.

The sign of the relative thermal strain can change along the crystal depth. Slope error amplitude is calculated by integrating the thermal strain. At low power, smaller strain occurs at the footprint than in the crystal bulk, resulting in concave bending. At medium power, the thermal expansion gradient is negligible and surface deformation is minimal. At high power, a large thermal expansion gradient causes a strong deformation with convex shape. Slope error increase with power is due to a stronger temperature gradient and thermal expansion coefficient. This explains the origin of the three deformation phases seen in previous studies (Zhang *et al.*, 2003 ▸, 2013 ▸; Huang *et al.*, 2014 ▸; Brumund *et al.*, 2021 ▸). The power and temperature regime at which the crystal is nearly flat, also called the ‘sweet spot’, should be carefully determined to prevent transition to the next regime, where deformation increases steeply with power.

## 3. Experimental study

### 3.1. Measurement details

Undesirable changes of the focused beam size at the end-station are observed on beamline I16. The vertical beam size is strongly affected by power levels on the crystal monochromator, which are in turn dependent on undulator settings, primary slits size and monochromator Bragg angle.

**Figure 6:**
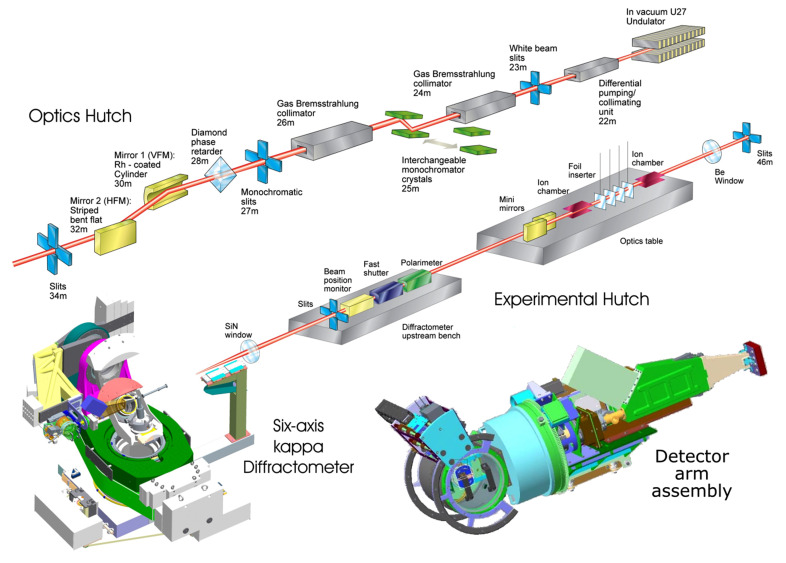
I16 beamline layout at DLS. The undulator radiation passes through primary adjustable slits and is nearly completely absorbed by the first Si crystal.

The optical layout is shown in Fig. 6 ▸. A 2 m-long in-vacuum undulator (IVU) source produces 3.2 kW total power at its minimum gap (5 mm). The beamline utilizes a set of primary slits (S_1_) at 23.6 m and a channel-cut Si(111) crystal monochromator at 25.5 m from the source. The crystal is cooled indirectly by flowing LN2 through two cooling channels inside the Cu block (Fig. 1 ▸). No filters or windows are used since the photon energy range extends to *E* = 2.5 keV. A set of vertical and horizontal focusing mirrors at 29.5 and 31 m, respectively, focus the beam at the sample position at 50 m from the source.

Beam size and photon flux at the sample position were measured for different values of incident power. The crystal temperature *T*_b_ was continuously recorded by a thermocouple attached to the crystal side. The synchrotron ring current was nearly constant, *I* = 300 mA (±1%, due to top-up), and the power was varied by varying the selected photon energy, the undulator gap values and the size of the primary slits S_1_, up to 5 mm × 3 mm. This multi-parameter approach gave detailed insight into the crystal response to power, compared with previous studies where ring current was the main variable (Zhang *et al.*, 2013 ▸; Antimonov *et al.*, 2016 ▸). Power scenarios, calculated using the *SPECTRA* code (Tanaka & Kitamura, 2001 ▸), are summarized in Table 1 ▸. Absorbed power could be up to 10% lower than theoretical values (Zhang *et al.*, 2013 ▸); however, this does not affect the following discussion.

### 3.2. Results and discussion

**Figure 7:**
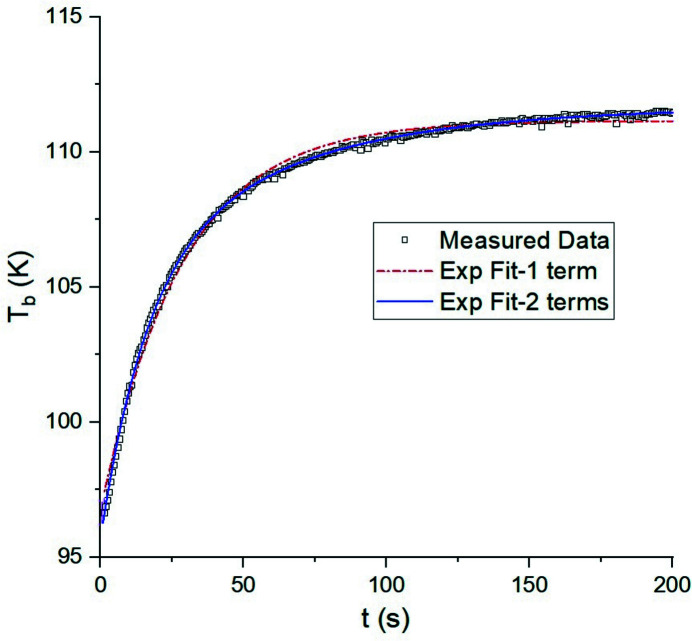
Temporal evolution of I16 crystal temperature for an undulator gap of 7.0 mm and S_1_ size of 2.5 mm × 1.0 mm. The red and blue lines are fit results with one and two exponential terms, respectively.

An example of the measured evolution of *T*(*t*) is shown in Fig. 7 ▸: temperature increases exponentially from 96 K to 112 K with a time scale of about 100 s. Data are well fitted by equation (3), even using the constant *T*_Cu_(*t*) approximation. Fitting parameters at two gap values are summarized in Table 2 ▸ and we obtain *k*_SiCu_ ≃ 1820 W m^−2^ K^−1^ for this crystal, using equation (11).

**Figure 8:**
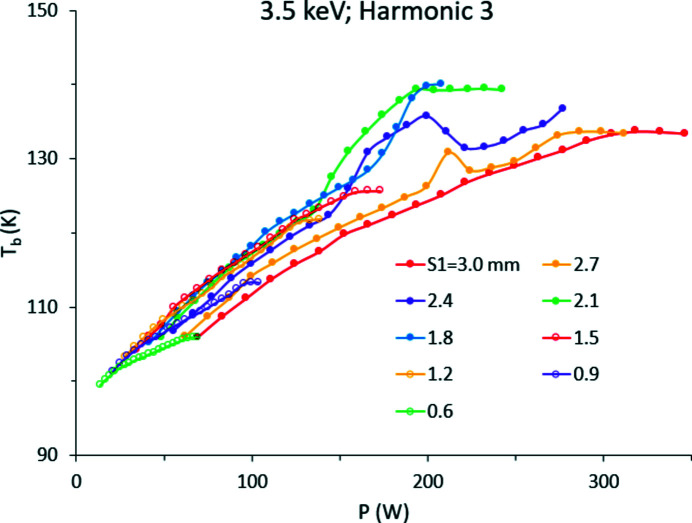
Steady state values of *T*_b_ versus calculated power for *E* = 3.5 keV and 5.0 keV and different slit size.

Contact conductance *k*_SiCu_ is an important parameter, which is sometimes estimated experimentally. Several studies have previously suggested that an initial analytical estimation, followed by a comparison of the measured temperatures in the actual conditions with the theoretical prediction, can be successfully used to determine the contact conductance with the required accuracy (Yovanovich, 2005 ▸). To design cryo-cooling systems, comparison of FEA with experiments should be used to reduce the uncertainty. Multiple measurements are necessary to determine an average value for *k*_SiCu_, as it often depends on *P* and *P*_d_. Alternatively, it can be calculated from the slope of *T*_b_ versus *P* as shown in Fig. 8 ▸. *T*_b_ increases approximately linearly at low to medium power (*P* < 150 W) and at smaller rate at higher power. Such a slower increase is probably due to higher cooling efficiency and increased effective Cu–Si contact area at higher *T*_b_. We obtained values ranging from *k*_SiCu_ ≃ 2500 W m^−2^ K^−1^ (*E* = 8 keV) to ∼3500 W m^−2^ K^−1^ (*E* = 3.5 keV), as in previous analyses (Zhang *et al.*, 2003 ▸, 2013 ▸).

**Figure 9:**
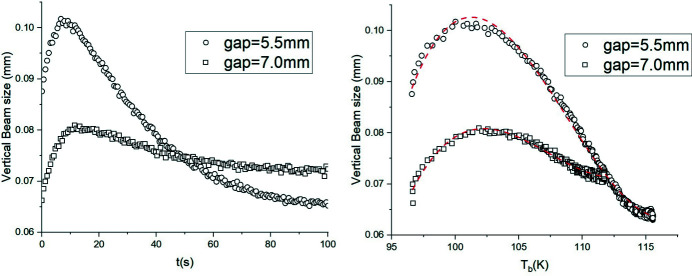
Vertical beam size versus time (left) and measured crystal temperature (right) for two gap values at *E* = 8.0 keV. The red dashed lines in the right-hand plot show the third-order polynomial fit of the data [equation (21)].

The experimental vertical beam size is proportional to crystal slope error θ, in good approximation. It has been measured *via* an imaging camera, *versus* time and *T*_b_, as shown in Fig. 9 ▸. It increases for about 10 s, to reach its maximum value, and then decreases to its final equilibrium value exponentially, when the crystal temperature reaches *T*_b_. The same trend is predicted by equation (21), which has been used to fit the data.

To understand the dynamic behaviour, a Python code was written, based on the heat equation. The I16 crystal monochromator temperature was calculated iteratively, showing that *T*_p_ reaches its maximum value in fractions of a second, with *T*_b_ static. Successively, both *T*_p_ and *T*_b_ increase at a similar rate, to reach a steady state, with time scales of about 1 min. Fig. 9 ▸ shows that the beam size changes continuously until it reaches the equilibrium state. This confirms that both *T*_p_ and *T*_b_ contribute to the surface deformation.

**Figure 10:**
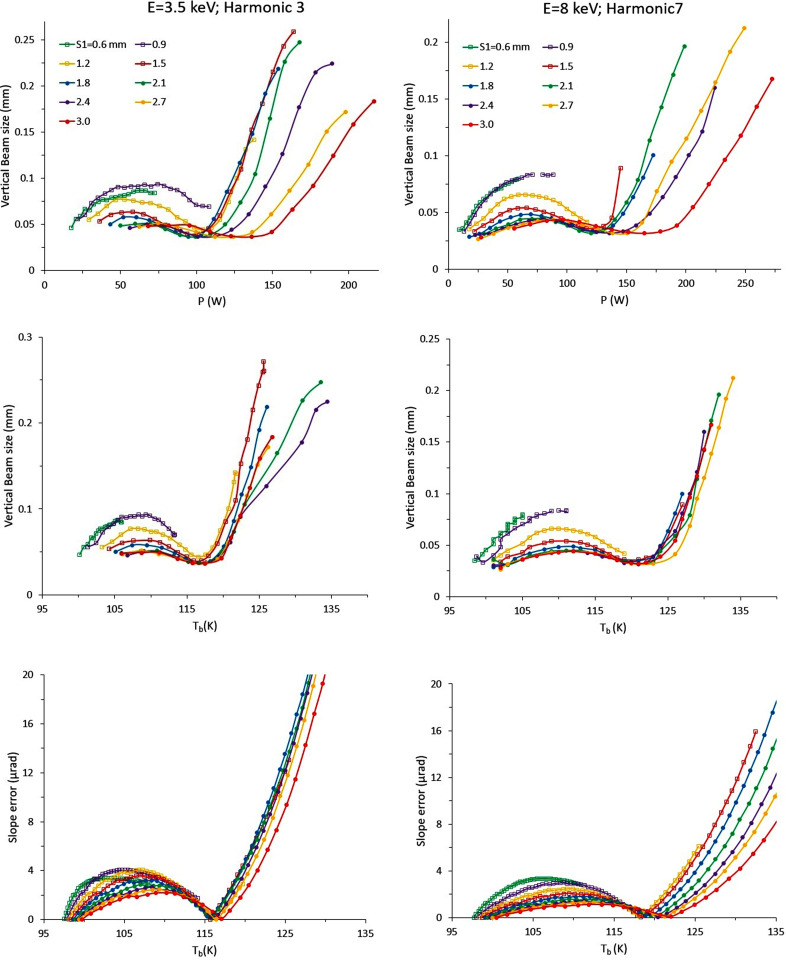
Measured vertical beam size at the focal position versus calculated power (top) and *T*_b_ (middle) for typical power conditions. Bottom: slope error from the model in Section 2 at *E* = 3.5 keV (left) and 8 keV (right). Legends indicate the vertical opening of S_1_.

Equilibrium values of vertical beam size versus absorbed power are shown in Fig. 10 ▸ (top). Local minima of the beam size are of order 20–30 µm, which is twice the value expected from modelling the monochromator as a flat perfect crystal. Different regimes are observed: a smooth increase is measured at low power; beam size then decreases at medium power values, reaching its minimum at the ‘sweet spot’; a steep increase to 200–400 µm with increasing the heat load. This is a critical scenario, presenting a beam-size linear trend of around an order of magnitude larger than at low power. In summary, crystal deformation does not simply increase linearly with absorbed power. Similar trends for the slope error are reported (Zhang *et al.*, 2003 ▸, 2013 ▸; Huang *et al.*, 2014 ▸; Brumund *et al.*, 2021 ▸; Huang & Bilderback, 2012 ▸), as this is a universal behaviour. In this study, critical values for the ‘sweet spot’ are in the range *P* ≃ 80 to 180 W depending on values of *P*_d_ and of incident beam size, whilst they are of order 300 W to 400 W in Zhang *et al.* (2013 ▸). The large difference is due to the very different cooling geometries.

A better parameter to control crystal deformation is the *T*_b_ temperature. The vertical beam size data in Fig. 10 ▸ (centre) indicate that the scenario of minimum deformation is achieved in the range *T*_b_ = 116 K (*P*_d_ = 11 W mm^−2^) to 121 K (*P*_d_ = 6 W mm^−2^). The slope error from the model (Fig. 10 ▸, bottom) is a good fit to experimental data. A cooling design with accurate feedback is suggested to minimize the deformation and prevent any transition to the very high deformation regime.

## 4. Conclusions

On-going upgrades of synchrotron sources and instrumentation deliver brighter and brighter beams of X-rays for applications ranging from biology to chemistry, from physics to engineering. Large investments in new sources are accompanied by progress in optics, detector technology and data storage. Study of matter at the nano-scale with X-rays, which was unthinkable when the first third-generation sources were built, is now reality. Stability and perfection of crystal monochromators is paramount to conserve the flux and brightness of synchrotron beams. This model of temperature and crystal deformation in indirectly cryo-cooled Si crystals illustrates and explains the dependence of crystal thermal slope errors observed in several synchrotron monochromator studies. Used in conjunction with FEA, it has been used to finalize crystal design and cooling at Diamond Light Source and to assess the resilience of these new optics for the future synchrotron machine.

## Figures and Tables

**Table 1:** Calculated average power density *P*_d_ at S_1_ and at the crystal surface for different values of photon energy and undulator deflection parameter *K*_IVU_

*E* (keV)	*K*_IVU_	Harmonic	*P*_d_ at S_1_ (W mm^−2^)	*P*_d_ on crystal (W mm^−2^)
3.5	1.85	3	23.1	11.2

5.0	2.08	5	26.2	8.9
	1.34	3	15.8	5.4

8.0	2.27	9	28.7	6.1
	1.87	7	23.3	5.0
	1.39	5	17.4	3.7
	0.61	3	6.1	1.3

**Table 2:** The fitted parameter for the single- and double-exponential models, using data in Fig. 7 ▸ Other constant (or average) values used are: *C*_Si_ (105 K) ≃ 260 J kg^−1^ K^−1^, *m*_Si_ = 0.21 kg, *T*_LN2_ = 82 K, *A*_SiCu_ = 15 cm^2^.

		One exponential term			Two exponential terms				
Gap (mm)	*P* (W)	*T*_b_	*T*_1_	τ_+_	*T*_b_	*T*_1_	τ_+_	*T*_2_	τ_−_
5.5	73	115	−18	33	115	−11	17	−8	59
7.0	60	111	−14	32	112	−10	17	−6	59

## Appendix A

### A1. Derivation of equation (3)

Equations (1) and (2) are combined to obtain a second-order differential equation for *T*,

where

All other parameters are assumed to be independent of temperature and averaged over time and space. In practical cases, *k*_SiCu_ includes a contribution from the heat conductance of the indium foil. *T*_Cu_(*t*) is assumed constant along the Cu–Si and Cu–LN2 contact surfaces.

Inserting equation (3) into equation (8),

Using equations (8), (9) and (10),

### A2. Derivation of characteristic temperatures

At low power, with *T*(*r*) slowly varying, *k*_Si_ can be assumed constant and the solution to equation (4) is

where

The solution is: *T*_p_ = *T*_b_ + *P*/(14.3*a* *k*_Si_), where *T*_p_ = *T*(0) is the peak temperature.

At high incident power,

where *T*_a_ = *T*(*r* = *a*) , Λ_1_ = 34.4 K, Λ_2_ = 158.4 K,  =  ;  =  , where γ = 1.6 × 10^−6^ m W^−1^, ɛ = 2.1 × 10^−6^ m W^−1^.

Typical average values are *P* = 200 W, *P*_d_ = 10 W mm^−2^ and *a* ≃ 2.5 mm at DLS. Equations (6*a*) and (6*b*) can be approximated as

Integration of equations (14) over the beam footprint and the remaining Si bulk,

where *P*_radial_(*r*) = (π*r* ^2^/4*a* ^2^)*P*. All parameters are in standard SI units. For a circular footprint, *A*(*r*) = 2π*r* ^2^; numerical integration is performed in the case of an elliptical footprint,

Here, *r*_1_ and *r*_2_ are the semi-major and semi-minor axes of the elliptical beam footprint. *k*_Si_ is assumed to be constant at low power, when temperature changes only slightly inside the crystal. However, at high power, one of the following functions can be used,

with *T* expressed in K, and *k*_Si_(*T*) in W m^−2^ K^−1^. These functions have been obtained from fitting the experimental data of Touloukian *et al.* (1971 ▸) in the range of interest 80 K to 180 K. Both forms can be integrated analytically; however, equation (15) leads to simpler expressions,

Use of the more accurate expression in equation (16) leads to

where

Therefore, *T*_p_ and *T*_b_ can be calculated for any cooling design.

### A3. Derivation of thermal strain

The temperature dependence of the Si thermal expansion coefficient (Middelmann *et al.*, 2015 ▸) in the range 80–240 K leads to the following expression,

with parameters obtained from data fitting: *A* = −1.5108 × 10^−13^ m K^−4^; *B* = 9.5288 × 10^−11^ m K^−3^; *C* = −1.2778 × 10^−8^ m K^−2^; *D* = −1.4974 × 10^−7^ m K^−1^; *E* = −1.7656 × 10^−4^ m.

At low power,

where *p* = *P*/(14.3 *a* *k*_Si_).

The  polynomial term in the slope error is observed experimentally.

## References

[bb2] Antimonov, M. A., Khounsary, A. M., Sandy, A. R., Narayanan, S. & Navrotski, G. (2016). *Nucl. Instrum. Methods Phys. Res. A*, **820**, 164–171.

[bb3] Brumund, P., Reyes-Herrera, J., Detlefs, C., Morawe, C., Sanchez del Rio, M. & Chumakov, A. I. (2021). *J. Synchrotron Rad.* **28**, 91–103. 10.1107/S1600577520014009 33399557

[bb4] Cheng, X., Zhang, L., Morawe, C. & Sanchez del Rio, M. (2015). *J. Synchrotron Rad.* **22**, 317–327. 10.1107/S1600577514026009 25723932

[bb5] Chumakov, A., Rüffer, R., Leupold, O., Celse, J.-P., Martel, K., Rossat, M. & Lee, W.-K. (2004). *J. Synchrotron Rad.* **11**, 132–141. 10.1107/S0909049503026785 14960777

[bb6] Collins, S. P., Bombardi, A., Marshall, A. R., Williams, J. H., Barlow, G., Day, A. G., Pearson, M. R., Woolliscroft, R. J., Walton, R. D., Beutier, G., Nisbet, G., Garrett, R., Gentle, I., Nugent, K. & Wilkins, S. (2010). *AIP Conf. Proc.* **1234**, 303–306.

[bb7] Dolbnya, I. P., Sawhney, K. J. S., Scott, S. M., Dent, A. J., Cibin, G., Preece, G. M., Pedersen, U. K., Kelly, J. & Murray, P. (2019). *J. Synchrotron Rad.* **26**, 253–262. 10.1107/S1600577518014662 PMC6337885 30655493

[bb8] Huang, R. & Bilderback, D. H. (2012). *Proc. SPIE*, **8502**, 85020B.

[bb9] Huang, R., Bilderback, D. H. & Finkelstein, K. (2014). *J. Synchrotron Rad.* **21**, 366–375. 10.1107/S1600577514000514 PMC3945422 24562557

[bb11] Kazimirov, A., Revesz, P. & Huang, R. (2007). *Nucl. Instrum. Methods Phys. Res. A*, **576**, 422.

[bb10] Kazimirov, A., Revesz, P. & Huang, R. (2008). *Proc. SPIE*, **7077**, 707702.

[bb12] Marot, G., Rossat, M., Freund, A., Joksch, S., Kawata, H., Zhang, L., Ziegler, E., Berman, L., Chapman, D., Hastings, J. B. & Iarocci, M. (1992). *Rev. Sci. Instrum.* **63**, 477–480.

[bb13] Middelmann, T., Walkov, A., Bartl, G. & Schödel, R. (2015). *Phys. Rev. B*, **92**, 174113.

[bb14] Revesz, P., Kazimirov, A. & Bazarov, I. (2007). *Nucl. Instrum. Methods Phys. Res. A*, **582**, 142–145.

[bb15] Rutishauser, S., Rack, A., Weitkamp, T., Kayser, Y., David, C. & Macrander, A. T. (2013). *J. Synchrotron Rad.* **20**, 300–305. 10.1107/S0909049513001817 23412487

[bb16] Tanaka, T. & Kitamura, H. (2001). *J. Synchrotron Rad.* **8**, 1221–1228. 10.1107/s090904950101425x 11679776

[bb17] Touloukian, Y., Powell, R., Ho, C. & Klemens, P. (1971). *Thermophysical Properties of Matter*, Vol. 1, *Thermal Conductivity – Metallic Elements and Alloys.*

[bb18] Yates, B., Hu, Y. & Nagarkal, V. (2010). *Proc. SPIE*, **7802**, 78020U.

[bb19] Yovanovich, M. M. (2005). *IEEE Trans. C. Packag. Technol.* **28**, 182–206.

[bb20] Zhang, L., Lee, W.-K., Wulff, M. & Eybert, L. (2003). *J. Synchrotron Rad.* **10**, 313–319. 10.1107/s0909049503012135 12824931

[bb21] Zhang, L., Sánchez del Río, M., Monaco, G., Detlefs, C., Roth, T., Chumakov, A. I. & Glatzel, P. (2013). *J. Synchrotron Rad.* **20**, 567–580. 10.1107/S0909049513009436 PMC3943555 23765298

